# Perceived value interviews and socio-economic survey data for communities in rural Uganda

**DOI:** 10.1016/j.dib.2021.107734

**Published:** 2021-12-18

**Authors:** Stephanie Hirmer, Alycia Leonard, Sofia Conforti, Costanza Conforti

**Affiliations:** a Energy and Power Group, University of Oxford, United Kingdom; b Rural Senses, Oxford, United Kingdom; c University of Bologna; d Language Technology Lab, University of Cambridge, United Kingdom

**Keywords:** Needs assessment, Value data, Perception, Beneficiaries, Developing countries, Sub-Saharan Africa

## Abstract

This article describes a dataset of perceived values and socioeconomic indicators collected in rural Ugandan communities. The data were collected in interviews which employed: (1) the User-Perceived Value game, which solicits verbal data using graphical prompts and ‘why’-probing; and (2) socio-economic surveys, which collected demographic data. The dataset constitutes 119 interviews conducted between 2014 and 2015 in seven rural Ugandan villages. Interviews were conducted in various settings (e.g. individual/group, women/men/mixed) and in seven different local languages (which were subsequently translated into English). These interviews were part of a research project aiming to better understand what is important to rural communities in Uganda, and to investigate decision-making as a function of different demographics. This dataset can be used by researchers and practitioners in various fields such as sustainable development (e.g. to analyze how development initiatives may be designed to match community values) and natural language processing (e.g. to automatically perform perceived value classification from the expert-annotated interviews).

## Specifications Table

**Table:** 

Subject	Social Sciences
Specific subject area	Planning and Development
Type of data	Json file, csv file, table and figure
How data were acquired	Face-to-face interviews consisting of: (a) User-Perceived Value (UPV) game transcripts [1] conducted in varying settings (individual, women/men/mixed focus groups), and semi-structured socio-economic survey
Data format	Raw and analysed
Description of data collection	Data was collected from households that had previously benefited from rural electrification access schemes.
Data source location	Country: Uganda Districts: Arua, Moyo, Bukwo, Kanungo, Kabarole
Data accessibility	In data repository. Repository name: Zenodo Data identification number: 10.5281/zenodo.5779234 Direct URL to data: https://zenodo.org/record/5779234#.Ybi35n3P1EI
Related research article	S.A.Hirmer, A. Mazzone, A. Leonard, C. Conforti, The power of language: Exploring values, empowerment dynamics and communication strategies for gender-inclusive energy service design in rural Uganda, Energy Res. Soc. Sci. 85:102379 (2022). https://doi.org/10.1016/j.erss.2021.102379.

## Value of the Data

• These data can be used to better understand what is important to rural communities in Uganda and identify potential factors for decision-making amongst varying demographics (e.g. gender, age, income-levels).
• These data can be useful to practitioners working on projects with rural and remote communities in Uganda (e.g. project developers, development workers), as well as to the academic and scientific communities.
• Furthermore, these data can be used to analyze the relationship between the perceived values and a number of other dimensions, including education, gender, interview setting, and many others as done by Hirmer et al. [2].
• Finally, the data can be used to train classifiers, such as deep learning-based models, to automatically perform the task of perceived value classification from the expert-annotated interviews, in a similar way as done by Conforti et al. [3].

## 1 Data Description

The dataset described in this article was collected in seven communities across Northern, Eastern, South-western, and Western Uganda between 2014 and 2015. It comprises information about household characteristics (including education and employment; living situation; infrastructure; household shocks, borrowing and household debt; subjective wellbeing and social attitudes; and religion and exposure), and personal values. The information on household characteristics were obtained from socio-economic surveys, and the data on values by interviewing community members using the UPV game in different settings. A codebook describing value labels for variables and the questionnaires that were used for data collection is also available to accompany this dataset. However, identifying variables such as names, GPS coordinates, and village names are anonymised.

The dataset is composed of two main parts: the qualitative UPV interviews and the socio-economic surveys. In the sections below, first we describe how the dataset was built, and then we describe each of these two main parts in detail.

## 2 Dataset Building

The dataset was collected in three main stages, summarised in Table 1.

**Table 1:** Setting specification at different stages of the dataset construction.

**Data Collection**	Timeline	Between 2014 and 2015
	Location	7 rural villages in different areas of Uganda.
	Collectors	Ugandan citizens fluent in the different local languages, who participated in a training workshop.
	Methodology	The interviews were conducted in locations familiar to the interviewees, mostly in open air. The interviews were conducted both individually and in groups of six following standard focus group methods. To avoid direct inquiry, the interviews were conducted by means of the *UPV game*, which is described in detail in [1], resulting in semi-structured interactions. Each interviewee also completed a survey.
**Data Translation**	Timeline	2015
	Location	Various villages in rural Uganda
	Translators	Ugandan citizens from the same areas where the interviews were collected. All translators spoke *Uglish* (the Ugandan variety of English) fluently as a second language.
**Data Annotation**	Timeline	2019
	Location	United Kingdom
	Annotators	A 30-year-old Bavarian woman native in German with fluency in English, who had worked and lived in rural Uganda prior to that work, and a 3rd year Ph.D. student in Natural Language Processing.
	Methodology	Each utterance was separately annotated. In case of disagreement, the final labels were decided through discussion.

## 3 Survey

### 3.1 Data format

The survey data is provided as a comma-separated values file (CSV), which reports the speakers’ answers to a set of 49 questions. In the file, each column represents a question, and each row represents a single speaker's answers. The questions are recorded in the CSV header (i.e. the first row).

To ease analysis, a mapping between the questions in the csv and all possible answers is provided as a single file in JavaScript Object Notation (JSON) format. The JSON file contains a dictionary of dictionaries, in which each dictionary represents a question and its possible values. This is illustrated by the example below, which reports the json entry for the question on ‘Education Status’.

**Table:** 

``Education Status'':{ ``1'':``Never enrolled'', ``2'':``Completed school'', ``3'':``Currently enrolled'', ``4'':``Drop out'' }

## 4 Data Description

The interviews and surveys were collected orally in the native language of the speakers. A complete list of survey questions is provided in Table 2. Speaker demographics are reported in Table 3. The data comes in an anonymized, disaggregated form.

**Table 2:** List of questions in the survey.

1	Sex?
2	Marital Status
3	# of Children (Note down age)
4	Relationship to other Participants (State Names of other participants if necessary)
5	Education Status
6	Highest grade completed in general education (# of years completed)
7	Does/Did attend any technical/vocational school or college, either privately run or publicly run?
8	What is the employment status of the participant?
9	What are the sources of income of your household? (In cash and in kind; can write multiple & role HH member i.e. son, husband/wife)
10	If ‘unemployed’, how would classify themselves?
11	What type of dwelling do the participants live in?
12	How many dwellings does your household own?
13	What rooms do the dwellings represent? (Incl. kitchen, dining room, lounge, & bedroom); (excl. bathroom/toilet/passages)
14	How many people live in your household?
15	What is the ownership of the dwelling?
16	How much agricultural land does your family own?
17	How much non-agricultural land does your family own?
18	Does the water used for drinking come from same source as the water used for other purposes like bathing or washing clothes?
19	How far does the household have to go (one way) to fetch water?
20	What is the source of water most often used in this household for drinking, bathing and washing clothes?
21	What kind of toilet does the household use?
22	What is the main source of lighting?
23	Is this household connected to electricity supply?
24	Is the system operational?
25	What is the electricity used for?
26	What appliances is the electricity used for?
27	What is the household's main source of energy for cooking?
28	Has anyone in the household suffered any serious injuries or illnesses in the past year?
29	Has the household suffered from any serious loss of assets/income through any of the following in the past year?
30	Has anyone in the household done the following in the last year borrowed money from:
31	How much has the household borrowed in total? (i.e. total debt owed to others)
32	What is the main purpose of current borrowing?
33	All things considered, how satisfied is the participant with life?
34	How satisfied is the participant with their financial situation?
35	All things considered, how satisfied is the participant with their current work?
36	When comparing well-being with other people, whom do you compare yourself with?
37	Religion of participant
38	How strong is the participant's belief in God?
39	Would say he/she is a religious person?
40	How often does the participant attend church/mosque/temple etc.?
41	Generally speaking would say that most people can be trusted or that one can't be too careful in dealing with people?
42	Has the participant been to any major city in Uganda?
43	Has the participant been to anywhere outside Uganda?
44	# of days stayed away from home in last year?
45	If stayed away for more than a month, what was the main reason for absence?
46	How often do you listen to the Radio?
47	How often do you watch TV?
48	How often do you read the newspaper?
49	How often do you use the internet?

**Table 3.** Demographic characteristics of the speakers.

Total speakers	84
Gender	Equal split women/men
Nationality	Mostly Ugandan
First language	One (or more) of the following: Rukonjo, Rukiga, Lugwere, and Swahili (Bantu family); Sebei/Sabaot, Kupsabiny, Lugbara (Nilo-Saharan family).
Age	From ∼15 (youngest) to ∼80 (eldest)
Socio-economic Status	Variable: political and religious leaders excluded, as well as close family members of the interviewee(s).

## 5 Qualitative Interviews

### 5.1 Data format

The perceived values dataset drawn from UPV game interviews is provided as a single file in JavaScript Object Notation (JSON) format. The file contains a list of 2,341 entries. Each entry contains the interview excerpt discussing one specific item, as part of the UPV game described in Section 2, and is encoded as a dictionary with the following specifications (Table 4).

**Table 4.** Specifications of an entry in the dataset.

key	value
speaker_id	*Description:* Code of the village in which the interview was collected *Type:* integer ∈ {1,...,84}
village_id	*Description:* Code of the village in which the interview was collected *Type:* string ∈ {*Northern Uganda, Eastern Uganda, Western Uganda, South-Western Uganda*}
interview_setting	*Description:* Setting in which the interview was collected *Type:* string ∈ {*single woman, single man, group woman, group man, mixed*}
item	*Description:* The item chosen as part of the UPV game *Type:* string
item_order	*Description:* Order in which the item was picked by the speaker *Type:* integer ∈ {1,...,20}
utterances	*Description:* Ordered list of utterances (i.e. transcribed spoken statements) pronounced during the UPV game when discussing the given item *Type:* list of strings
upvs_per_utterances	*Description:* Ordered list of lists, where each list at position *i* reports the UPV annotations for the *i*th utterance. *Type:* list of lists of strings

The following entry exemplifies this data format:

**Table:** 

{ “item'': ``computer'', ``item_order'': 9, ``speaker_id'': 71, ``village_id'': ‘South-Western Uganda’, ``interview_setting'': ``single woman'', ``utterances'': [ ``And it again helps in making work easy for example me; I take a knife to be a computer anywhere and anything that makes work easy I refer it as a computer.'', ``If I have a computer haaa I would be making money every minute because everyone here in <PLACE> wants to type work or print has to go <PLACE> for that computer work and if I had it I would do it from her not even putting transport and time.'', ``Also if I have a computer it can help my children to pass their exams, actually they can be the best in computer science because all the time they are practicing how to use a computer.'' ], ``upvs_per_utterances'': [ [``Memorability'', ``Unburden''], [``Time Benefit'', ``Capital Expenditure'', ``Availability'', ``Unburden'', ``Economic Opportunity''], [``Aspiration'', ``Family Caring'', ``Knowledge Attainment'', ``Personal Performance''] ] }

## 6 Data Description

**Fig. 1:**
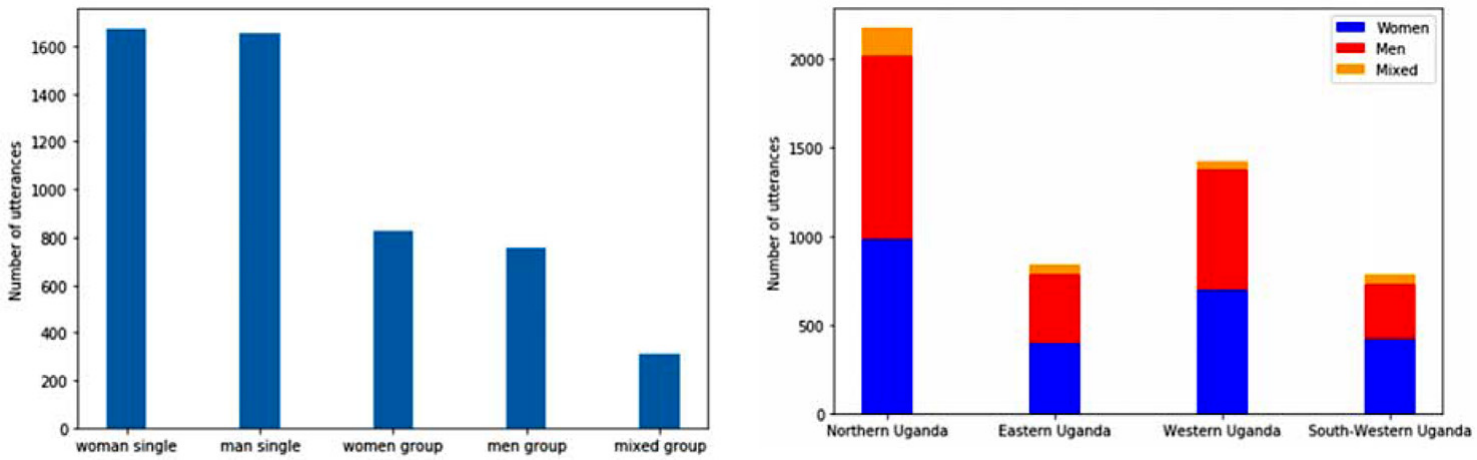
a. Number of utterances per interview setting in the dataset. b. Number of utterances per geographic area, disaggregated by speaker's gender.

The quantities of utterances are quite even between men and women and amongst the seven villages. The distribution of utterances per interview setting and per village in the dataset are reported in Fig. 1a and b.

**Fig. 2:**
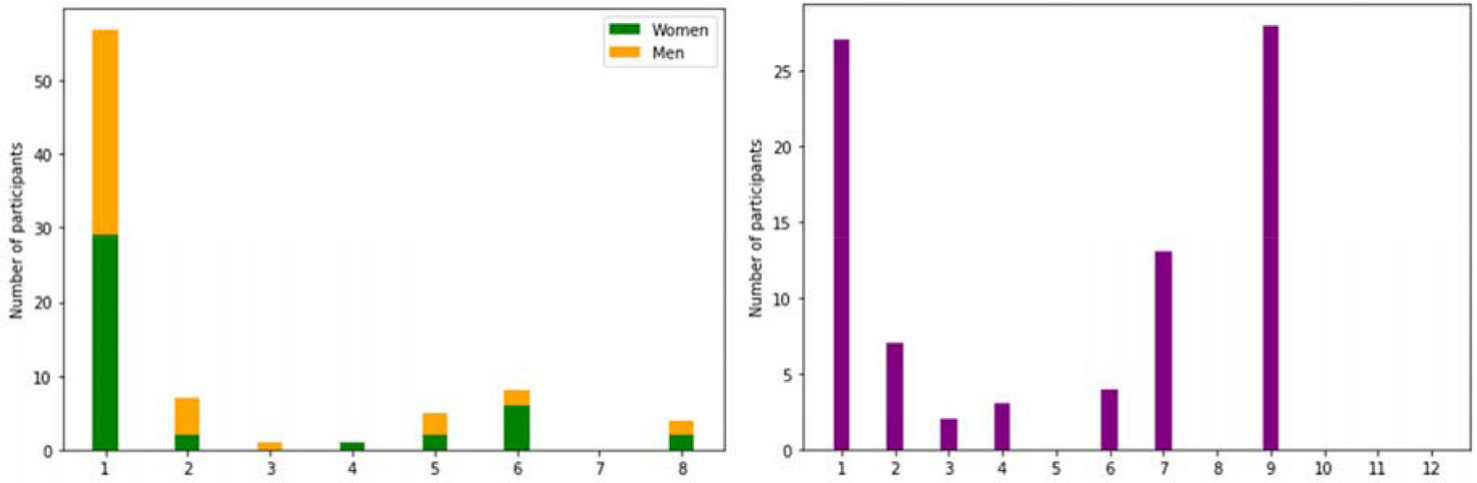
a. Distribution of employment of the speakers, disaggregated by gender. Legend: 1: Own farming activities, 2: Casual Labour in Agriculture, 3: Casual Labour non-Agriculture, 4: Salaried Employment in Agriculture, 5: Salaried Employment in non-Agriculture, 6: Petty business/trade/manufacturing, 7: Major business/trade/manufacturing, 8: Other. b. Distance to a source of water of the speaker. Legend: 1: Piped public tap/kiosk (free), 2: Piped public tap/kiosk (paid for), 3: Piped internal, 4: Piped tap outside, 5: Water carrier/tanker, 6: Well (non borehole), 7: Borehole/ hand pump, 8: Rainwater tank, 9: Flowing river/stream, 10: Dam/stagnant water, 11: Protected spring, 12: Others.

Fig. 2 reports the distribution of speakers over some of the considered variables (source of employment, and distance to a source of water).

While English is the national language in Uganda, the written English of translators was poor at times. As a consequence, the dataset contains some grammatical errors. Given that the interviews were manually transcribed, some utterances contain typos and spelling errors.

To protect the participants’ identity, the exact names of the villages are not released. All proper nouns (people, tribes, locations, ...) in the utterances are anonymized with special tags (such as <PERSON> or <LOCATION>).

**Fig. 3.**
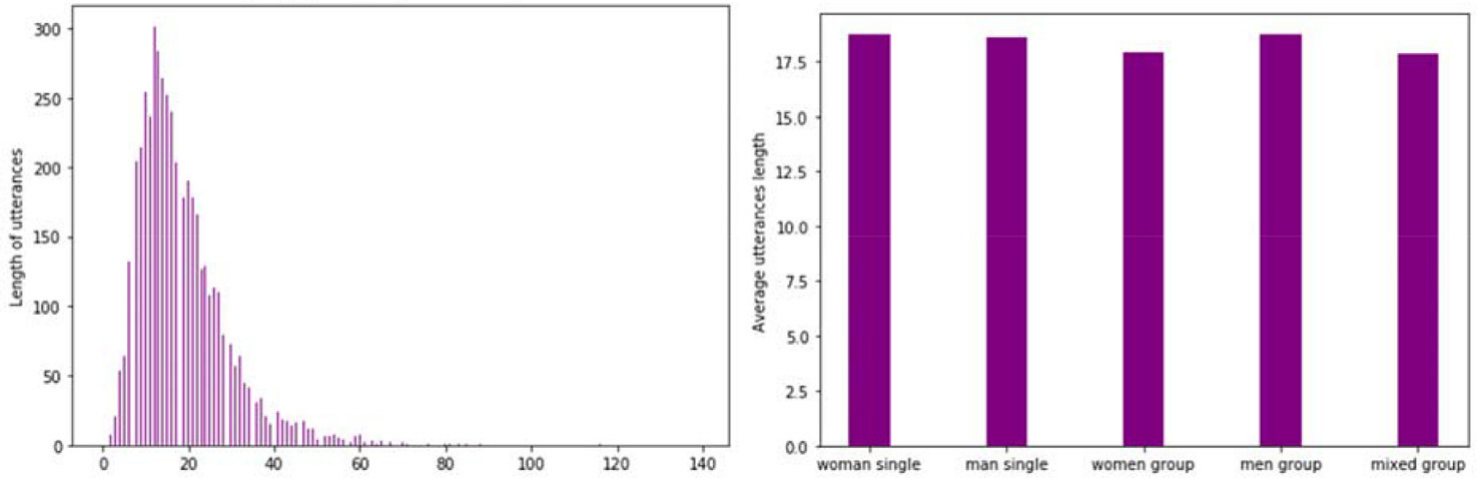
a. Distribution of utterances length (in words) in the dataset. b. Average utterance length (in words) per interview setting in the dataset.

On average, utterances are 18.5 words long (Fig. 3a), without any major difference between interview setting type (Fig. 3b).

## 7 Experimental Design, Materials and Methods

### 7.1 Sampling

In this section, the specific sampling considerations are described. A number of samples from specific populations were taken at different stages of the research for case selection, focus group members, and interviewees.

### 7.2 Case selection

Case villages were selected with the aim to provide a representative sample of a population of rural Uganda that could be interviewed. As these data were originally collected as part of a research project focusing on off-grid energy access, selected case villages must have benefited from off-grid energy access initiatives. Seven case study villages located from four regions of Uganda were selected. The regions included: Northern, Eastern, South Western, and Western Uganda.

### 7.3 Participant sampling

In each of the seven villages, 17 interviews were conducted with 12 participants (i.e. a total of 119 interviews and 84 participants). The sampling criteria listed in Table 5 were applied for both individual and focus group interviews. Note that the same people participating in the UPV game were also asked to take part in the socio-economic survey.

**Table 5.** Specifications of an entry in the dataset.

Category	Participants should consist of ...
Age	… varying age groups (18+)
Gender	… either male or female^1^
Social	… variety of social classes
Residency	… have lived in the village prior to the construction of the off-grid energy access project
Available	… be available the entire lengths of the session
	**Focus groups should not consist of…**
Relatives	… close relatives
Leaders	… leaders of organisations or governmental officials
Aggressors	… dominant or overpowering individuals

1 In this paper, gender refers to men and women as cisgender, meaning that both men and women identify themselves with the sex assigned at birth and embrace the notion of masculinity and femininity and their relative roles in accordance with the local context [2].

Five focus group meetings were held in each of the seven case villages with six participants in each group, giving a total of 35 focus groups. Each village focus group was initially separated into male and female participants to facilitate equal gender participation (as female participants in the rural Ugandan village setting may be less likely to voice opinions in the presence of male participants [4]). Members were selected to represent the diverse interests of the village, e.g. large family, farmer or female head of household [5]. It made use of the standard focus group method of a group interview, consisting of six participants [5], [6], [7].

### 7.4 Setup

Translators were employed to undertake the data collection with rural villagers in Uganda. This was necessary as in the four different regions containing the case villages, seven different languages were spoken. To ensure consistency in the collected data, a two-day training session was held with the translators prior to the fieldwork. During field interviews, each translator took notes and audio recordings were also made. To further ensure consistency across all villages, a local research assistant was hired to accompany and oversee all fieldwork.

Each participant (i.e. interviewee) received a small financial compensation of UGX 5,000 (£1.25). This roughly corresponds to the daily salary in case villages at the time of the interviews.

### 7.5 Narrative UPV game

The UPV game can help to better understand what users of development initiatives find to be of value. It requires participants to select items based on which are most important to them. Participants also have the option to identify new items or matters of value to them. This is followed by inquiry as to why the selected items were important. This approach is based on methods commonly used in market research and product design.

This method is designed to bypass interviewee predispositions and preconceptions by redirecting focus to the game itself. This focus helps prevent interviewees from trying to assume or second-guess responses. It encourages semi-structured storytelling and discussion of topics which resonated with each participant's experience.

The game seeks to identify user-perceived values (UPVs) which illuminate the underlying reasoning as to why selected items are important to beneficiaries. Such values are insufficiently captured by other needs assessment tools.

To gather the data described in this paper, the UPV game was played with participants in each of the seven villages. Participants were equally split between men and women and came from a variety of backgrounds and ages. The interviews were conducted in a variety of settings to obtain results representative of the community given the complex nature of influences on decision-making. The values, opinions, and preferences people express may vary in different group settings [8]. In light of this, there were 12 individual interviews and five group interviews per village. Group interviews included six participants each, and were held for:

• Men;
• Women;
• Mixed-gender (with the three most active participants from the previous discussions);
• Men discussing solutions proposed by women;
• Women discussing solutions proposed by men.

For each round of interviews (except when men/women discussing womens’/mens’ choices), participants were asked to (individually or as a group):

• **Select** 20 out of the 46 presented items based on what is important to them. Items included everyday products or services found in rural Uganda, such as livestock (e.g. cow, chicken), basic electronic gadgets (e.g. mobile phone, television, radio), household goods (dishes, soap, blanket), and horticultural items (e.g. plough, hoe). Participants could also name additional items they perceived as important. Items were depicted graphically to account for the low level of literacy across developing countries [9] like Uganda, where 43% are illiterate and rural areas are the worst affected [10].
• **Rank** their selection in order of importance.
• **Give reasons** as to why these items are most important to them personally. At this stage, participants were encouraged to give reasons (“why is this important to you?”) that reflected their personal lives. This method is called “why-probing”. Answers were in the form of storytelling.

### 7.6 Narrative socio-economic survey

To contextualise the data gathered from the UPV game, a socio-economic survey was also conducted. This data can be used to understand the participants’ stories and value perceptions within the broader context of their lives, demographics, and status.

The socio-economic survey was conducted in a semi-structured way to allow for flexible dialogue rather than rigid questioning [5]. Similar to the UPV game, the interviews were conducted with help of translators in local languages.

The socio-economic survey was conducted during the same visit as the UPV game with the same 12 participants in each village. It collected data within the following categories: general information; education and employment; living situation; infrastructure; household shocks, borrowing and household debt; subjective wellbeing and social attitudes; and religion and exposure (see Table 2). In addition to providing background information, the survey gathered information on life stage, external influences, and social, economic and cultural predispositions and perceptions.

## Ethics Statement

To ensure the study's integrity, a risk and ethics assessment following the Cambridge School of Technology Research Ethics Committee at the University of Cambridge in accordance with the procedures laid down by the University for Ethical Approval for all research involving human participants was completed and approved with Reference: R68195/RE001. To protect the participants’ identity, all names were removed.

## CRediT Author Statement

**Stephanie Hirmer:** Investigation, Methodology, Visualisation, Writing – original draft; **Costanza Conforti:** Software, Formal analysis, Visualisation, Writing – original draft; **Alycia Leonard:** Data curation, Writing – review & Editing; Sofia Conforti: Data curation

## Declaration of Competing Interest

The authors declare that they have no known competing financial interests or personal relationships which have or could be perceived to have influenced the work reported in this article.
